# The BEL information extraction workflow (BELIEF): evaluation in the BioCreative V BEL and IAT track

**DOI:** 10.1093/database/baw136

**Published:** 2016-10-01

**Authors:** Sumit Madan, Sven Hodapp, Philipp Senger, Sam Ansari, Justyna Szostak, Julia Hoeng, Manuel Peitsch, Juliane Fluck

**Affiliations:** 1 Fraunhofer Institute for Algorithms and Scientific Computing, Schloss Birlinghoven, Sankt Augustin, Germany; 2 Philip Morris International R&D, Philip Morris Products S.A, Quai Jeanrenaud 5, Neuchâtel, 2000, Switzerland

## Abstract

Network-based approaches have become extremely important in systems biology to achieve a better understanding of biological mechanisms. For network representation, the Biological Expression Language (BEL) is well designed to collate findings from the scientific literature into biological network models. To facilitate encoding and biocuration of such findings in BEL, a BEL Information Extraction Workflow (BELIEF) was developed. BELIEF provides a web-based curation interface, the BELIEF Dashboard, that incorporates text mining techniques to support the biocurator in the generation of BEL networks. The underlying UIMA-based text mining pipeline (BELIEF Pipeline) uses several named entity recognition processes and relationship extraction methods to detect concepts and BEL relationships in literature. The BELIEF Dashboard allows easy curation of the automatically generated BEL statements and their context annotations. Resulting BEL statements and their context annotations can be syntactically and semantically verified to ensure consistency in the BEL network. In summary, the workflow supports experts in different stages of systems biology network building. Based on the BioCreative V BEL track evaluation, we show that the BELIEF Pipeline automatically extracts relationships with an F-score of 36.4% and fully correct statements can be obtained with an F-score of 30.8%. Participation in the BioCreative V Interactive task (IAT) track with BELIEF revealed a systems usability scale (SUS) of 67. Considering the complexity of the task for new users—learning BEL, working with a completely new interface, and performing complex curation—a score so close to the overall SUS average highlights the usability of BELIEF.

**Database URL:** BELIEF is available at http://www.scaiview.com/belief/

## Introduction

To study the complex mechanisms of biological systems, network-based approaches such as the use of protein– protein interaction, metabolic, signaling, regulatory or coexpression networks are emerging in the field of systems biology (1–3). A computational representation of knowledge in a well-defined structured and standardized language is required for systematic network analysis. Unfortunately, this network information is often not available as structured data but can only be found as text in scientific publications. Biocuration is the process of translating biological findings and information to a formal and structured representation of biological data. The extraction of such findings from the scientific literature is an important and time-consuming task (4).

To handle the large volume of emerging and published literature and accelerate the curation process, biocurators need support from automated mining systems (4). Text-mining-assisted biocuration systems that extract information from the scientific literature represent one of several examples of such systems. Although a number of information extraction tools are already available, none of these tools directly outputs relationships in a network-modeling language. The development and availability of such text mining solutions raised the question whether the current state-of-the-art tools can be integrated and used in a workflow to support biocuration of networks. In this article, we present BELIEF as a workflow that focuses on knowledge extraction from literature in the biomedical domain while integrating various available state-of-the-art solutions. The workflow uses a text mining pipeline to extract relationships from literature. In the current state, we focus on sentence-based extraction of protein–protein interactions and relations between genes/proteins, chemicals, diseases and biological processes.

As a network modeling language, we use Biological Expression Language (BEL) (5) to represent the extracted knowledge. The information extraction system translates the extracted relations directly into BEL statements (BEL encoded triples). The web-based curation interface visualizes the causal and correlative BEL statements and facilitates the biocuration.

In addition to the system description, we present a detailed evaluation of the text mining components using the BioCreative V BEL track task 1 dataset (6, 7). We also describe the results of our participation in the BioCreative V Interactive task (IAT) track, in which several curators evaluated the usability of BELIEF by performing a biocuration task. Finally, we analyze and discuss the findings, summarize the lessons learned from this work, and consider the outlook for future releases.

### The modeling language BEL

The two most common network-modeling languages in systems biology are Biological PAthway eXchange language (BioPAX) (8), a format optimized for database exchange, and Systems Biology Markup Language (SBML) (9), a structured XML-based language. Although both have been used to model biochemical reaction networks, they have the drawbacks of not being designed for human readability. They also have restricted abilities for representing causal relationships to biological processes and diseases that are important in the understanding of disease mechanisms.

**Figure 1:**
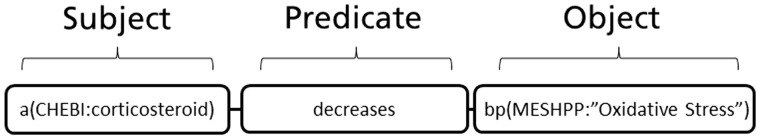
Structure of a BEL statement. This example of a BEL statement describes that an abundance of the chemical *corticosteroid* reduces the biological process *Oxidative Stress*. For the identification and disambiguation of domain-specific terms and concepts in BEL pre-defined namespaces, in this example, CHEBI and MESHPP are used. The namespaces CHEBI contains chemical entities from the resource ChEBI (http://www.ebi.ac.uk/chebi/) and MESHPP contains various biological processes from MeSH (http://www.nlm.nih.gov/mesh/) Phenomena and Processes [G] branch.

BEL was designed and engineered to drive biological network-based analytics (5, 10). BEL conserves the causal and correlative biological relationships gathered from the scientific literature with contextual and provenance information in a computable form. The relationships, also referred to as BEL statements, are triplets that can be decomposed into a subject, a predicate, and an object (Figure 1). Additionally, a BEL statement can be associated with context annotations that include citation information, the supporting evidence such as a text excerpt, and various experimental parameters such as cell line, cell structure and organism for the scientific observations. A detailed description of BEL syntax can be found elsewhere (11). An assembly of many BEL statements produces a causal network knowledgebase that can be used to understand and analyze the underlying biological mechanisms (12). In the past, trained users have manually extracted BEL statements and constructed network models through the process of biocuration. For example, several network models were used to understand the cause-and-effect mechanisms of pulmonary and vascular systems (13, 14). The BEL language and the BEL framework that integrates the language parser and the toolkits to create network models are part of the open source project named OpenBEL^1^.

### Overview of state of the art text mining systems

*BioCreative*^2^ is a community-wide effort initiated in 2003 that develops and evaluates information extraction systems. It focuses on text mining workflows and biocuration interfaces applied to the field of biology. Several BioCreative workshops that were previously held focused on various tasks, such as the identification of gene names and their normalization, detection of protein–protein interactions, extraction of chemical entities as well as the efficiency and the usability of biocuration interfaces (15–20). Several studies that used text mining for assisted manual curation have reported improvements. Textpresso represents such a real-world text mining application that was used to curate experimentally determined subcellular localization of *Caenorhabditis elegans* proteins while increasing curation efficiency by 8- to 15-fold compared with manual curation (21). In recent years, it had been adapted to further meet the needs of other model organism databases and incorporated into their extraction workflow (4). In another study, Tripathy *et al*. successfully used a text mining approach to extract information on the electrophysiology of neurons and their properties from the literature. They created the NeuroElectro database using the obtained results (22). Other text mining techniques were applied to extract context information for the most frequently annotated post-translational modifications in UniProtKB (23). The named entity recognition (NER) software ProMiner (24) was used to detect genes and proteins in the MGI biocuration workflow while increasing the curation efficiency by 20–40% without compromising on curation quality (25). Furthermore, Szostak *et al*. (26) successfully used BELIEF itself in the past to create an atherosclerotic plaque destabilization network. All together, these studies show that the text mining-assisted curation supports the biological network model building.

## System Description and Functionalities

**Figure 2:**
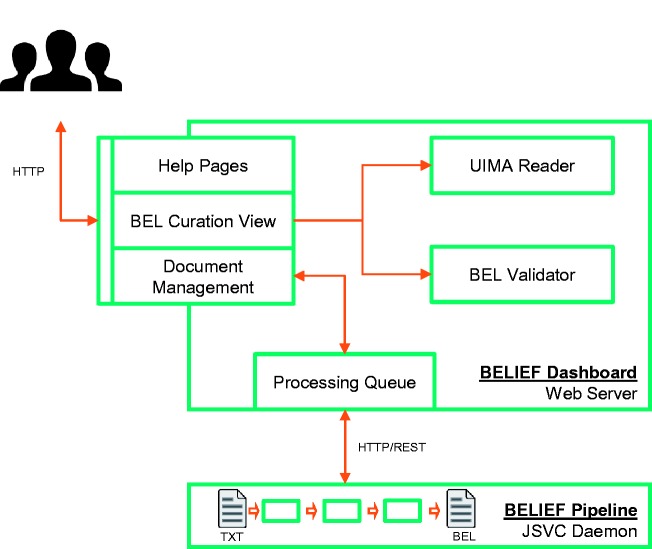
Architecture of semiautomatic information extraction workflow BELIEF. The workflow consists of a text mining pipeline (BELIEF Pipeline) and a web-based biocuration tool (BELIEF Dashboard). (*Note*: UIMA: Unstructured Information Management Architecture. UIMA Reader: A reader component to parse and extr act information from UIMA XCAS documents. JSVC Daemon: A Java library that allows applications to run as daemons.).

The BELIEF workflow contains two main components: the text mining pipeline (BELIEF Pipeline) and the web-based curation interface (BELIEF Dashboard) (Figure 2). Both components communicate through an HTTP/Representational State Transfer (REST) application-programming interface (API).

### BELIEF Pipeline

The BELIEF Pipeline consists of several components from the fields of natural language processing (NLP), NER and relationship extraction (RE) implemented in the Unstructured Information Management Architecture (UIMA) framework^3^. The full workflow is constructed as a non-interactive server application (based on commons daemon library^4^) to support text processing on demand. The workflow also includes a REST^5^-based BELIEF service that periodically contacts the BELIEF Dashboard via a defined API to pull new unprocessed documents into the RE workflow. The input data for the text mining workflow is the document text itself and the respective output data are the identified named entities and relationships. Hence, the runtime of the workflow strongly depends on the number of identified named entities and the document length.

**Figure 3:**
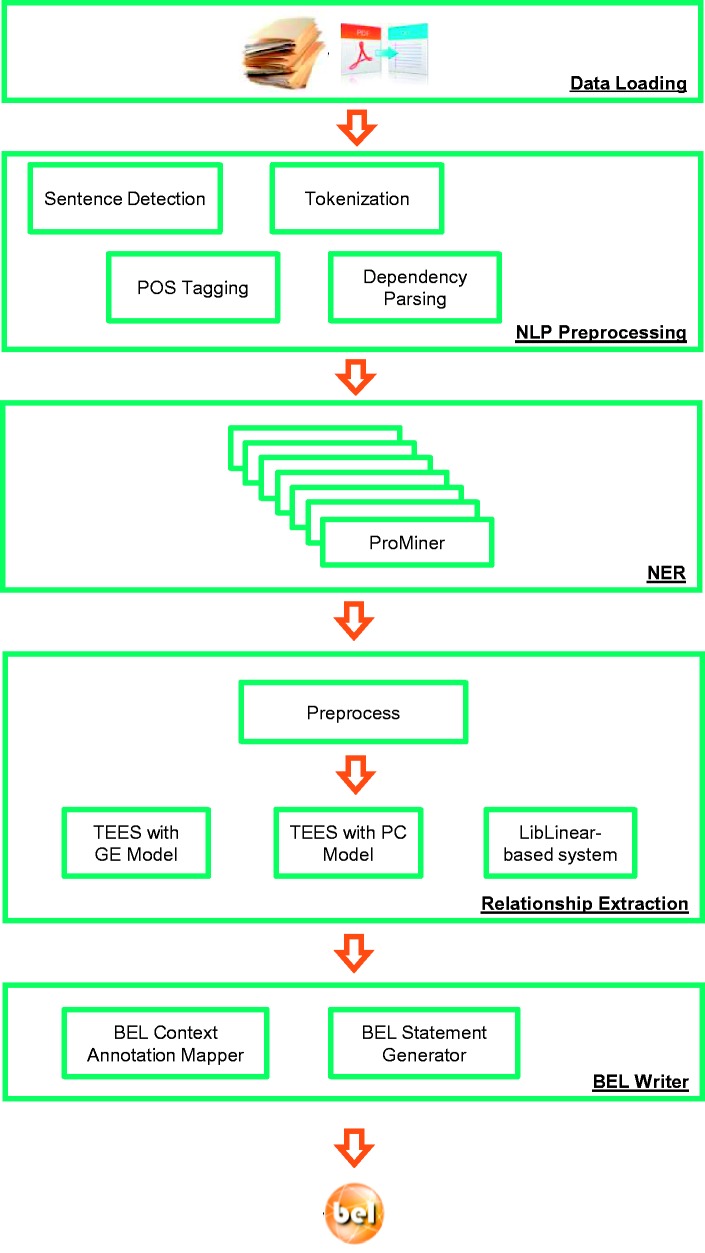
Architecture of the BELIEF text mining pipeline. (*Note*: POS Tagging: Part of Speech Tagging. NLP: Natural Language Processing. TEES: Turku Event Extraction System, a state-of-the-art relation extraction system. GE: Genia Event Extraction for NFkB knowledgebase, a BioNLP Shared Task. PC: Pathway Curation, a BioNLP Shared Task.).

Overall, the pipeline consists of several sequential minor and major steps (Figure 3). Owing to the flexible underlying UIMA architecture and the Common Analysis Structure (CAS) exchange format, the integration of new tools is simplified. A high level of modularity is required for the integration of new RE applications since several pre- and post-processing steps are necessary. Some of the pre-processing steps, such as the tagging of sentences or NER, can be shared by different tools. In this way, the duplication of work is prevented and runtime is optimized. The following sections describe the pipeline (Figure 3) in detail.

#### Text data loading

The input for the text mining workflow is text in various defined formats. There are different readers available to parse a variety of text formats, such as Medline abstracts and full text articles, both in Extensible Markup Language (XML). Alternatively, Portable Document Format (PDF) as well as plain text in UTF-8 encoding (8-bit Universal Character Set Transformation Format) is accepted. The current demonstration server of the BELIEF Dashboard only features the plain text reader. The text extractions can be started either from the command line or directly from the BELIEF Dashboard user interface. As input, the pipeline accepts single text files as well as a set of text phrases sent as a batch.

#### NLP pre-processing

Most of the subsequent steps in the workflow rely on appropriate pre-processing of free text. Therefore, several tools for sentence detection, tokenization, part-of-speech tagging and dependency parsing are integrated.

#### Named entity recognition and integrated dictionaries

One of the essential steps in this workflow is the NER with controlled vocabularies. Currently, the workflow incorporates ProMiner NER (27) that allows the normalization and integration of different vocabularies. ProMiner is well established for NER tasks and performs well in the recognition of gene and protein names (24, 28) as well as disease names (29). For the generation of syntax-valid BEL statements, it is necessary to establish mappings between NER annotations and existing namespace concepts or define new namespaces accordingly. Existing resources such as the ProMiner gene/protein name dictionaries and the MeSH disease dictionary were mapped to corresponding OpenBEL namespace identifiers and names, respectively. Other namespace resources, for example the OpenBEL protein family names, were extended with the most common synonyms. For chemical names, three resources (Table 1) were combined to provide greater coverage of entities.

**Table 1:** Used resources with the corresponding entity classes as well as the namespace symbols provided within OpenBEL

Entity class	Resources	OpenBEL namespace
Human genes/proteins	EntrezGene/Uniprot	HGNC
Mouse genes/proteins	EntrezGene/Uniprot	MGI
Rat genes/proteins	EntrezGene/Uniprot	RGD
Protein family names	OpenBEL	SFAM
Protein complex names	OpenBEL	SCOMP
Protein complex names	Gene Ontology	GOCC
Biological processes	Gene Ontology	GOBP
Chemical names	OpenBEL	SCHEM
Chemical names	ChEBI	CHEBI
Chemical names	ChEMBL	CHEMBL
Disease names	MeSH	MESHD
Anatomical names	MeSH	MESHAnatomy
Cell lines	Cell Line Ontology	CellLine
Cell structures	MeSH	CellStructure

These resources were converted into dictionaries and integrated into the workflow: EntrezGene (http://www.ncbi.nlm.nih.gov/gene), Uniprot (http://www.uniprot.org/*)*, OpenBEL name spaces and annotations (http://resources.belframework.org/belframework/), Gene Ontology (http://www.geneontology.org/), ChEBI (http://www.ebi.ac.uk/chebi/), ChEMBL (https://www.ebi.ac.uk/chembl/*)*, MeSH (http://www.nlm.nih.gov/mesh/), and the Cell Line Ontology (cellontology.org/).

**Figure 4:**
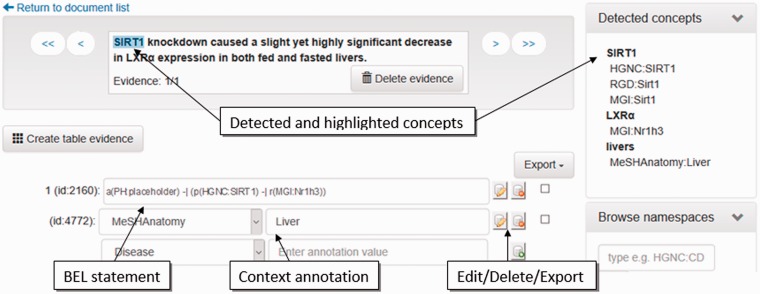
Screenshot of the evidence-centric curation view. In the upper left, the evidence text is visualized. Detected concepts in the current evidence text are shown in the upper right. In the bottom left, the curation of BEL statements and their context annotations can be performed.

Table 1 lists all dictionaries currently incorporated into the workflow, the corresponding entity classes, and the original resources, as well as the namespace symbols used within BEL. The recognized named entities can be used either as input for RE or as additional context annotations within the BEL document. Currently, all protein, gene, protein family, and protein complex names as well as chemical names and disease names are used as input for RE (‘Relationship extraction’ section). The anatomy, cell line and cell dictionaries are used for context annotations. These annotations are directly imported into the corresponding BEL document by the context annotation mapper and visualized as such in the curation interface (Figure 4, context annotation).

For biological processes, only a small part of Gene Ontology (GO) biological processes used in the BioCreative V BEL track is integrated into the RE process. The previous BioCreative IV Gene Ontology task has shown that the recognition of biological processes and their relationship with genes is very challenging (30). The most successful teams have achieved F-scores of around 0.134. In addition, our current RE system is not trained to extract relationships to biological processes. Therefore, for the biological processes not used in the BioCreative BEL task, we do not extract BEL statements. Instead, we show the detected concepts in the respective concept section of the curation interface.

#### Relationship extraction

Here, we have integrated two RE methods building on pre-annotated named entities. Because the NER modules perform independent annotations for each vocabulary, it is necessary to unify and harmonize overlapping matches. This task was combined into a separate UIMA component named the RE pre-process. For overlapping matches with different boundaries, the longest match is taken into consideration. A ranking scheme for hits that exactly overlap is given if an organism name is mentioned in the text in which this entity type is used; otherwise, a pre-defined ranking is used (e.g. HGNC over MGI or RGD). This arbitrary selection by ranking is only necessary for the subsequent RE tools. An example is given in Figure 4. The curation interface shows all detected entities to the user (Figure 4, upper right, detected concepts). In the upper left of the curation interface, the supporting evidence text is visualized. In this example, SIRT1 has been recognized as human, mouse, and rat gene. LXRalpha has been assigned to a mouse (MGI: Nr1h3) and a rat gene (RGD:Nr1h3). Furthermore, livers have been recognized as MeSHAnatomy term.

In the lower part of the curation interface, an automatically extracted statement is shown. This can be corrected by the human curator. False entities can be easily replaced by the entities from the detected concept part. In the example shown, the relationship is displayed between a human and a mouse gene. Through a copy and paste action, the human gene HGNC:SIRT1 can be easily replaced by the correct concept MGI:Sirt1. In future versions, we will implement a better disambiguation process for the selection of a more correct organism assignment.

In the example given in Figure 4, the anatomy concept liver is correctly placed as context annotation. The curator can select further context classes and their corresponding entities in the drop down menu.

One of the two RE methods is the linear support vector machine classifier LibLinear (31) that was trained on five publicly available training corpora [AIMed, BioInfer, IEPA, HRPD50 and LLL 05 generated by Pyysalo *et al*. (32)]. This approach uses lexical features such as bag-of-words and n-grams-based features. Additionally, dictionary-based domain-specific trigger words as well as dependency-parsing-based features are taken into account (33). The classifier takes the sentences with pairs of co-occurring ProMiner entities as input and returns the relationship information for the two entities. For the LibLinear classification, manual curation is required to improve the quality of the BEL statements. The classifier does not provide a predicate or the direction of the relationship. As a result, either the first or the second entity might be the subject of the relationship.

The Turku Event Extraction System (TEES) (34) nearly addresses all BioNLP shared tasks and is one of the top scoring tools for such (35). In the BELIEF workflow, TEES 2.1 including models trained on the Genia Event Extraction for NFkB knowledgebase (GE) and the Pathway Curation (PC) was integrated as a single UIMA component. For further details of these tasks, we refer the reader to the BioNLP shared task webpage^6^ (36). The annotated text with all relevant named entities acts as an input for TEES. In its standard implementation, TEES uses the NER module BANNER (37) for entity recognition, but BANNER does not normalize identified entities to concepts. Thus, the TEES internal named entity recognizer BANNER was replaced by ProMiner. In the BELIEF workflow, the GE model processes protein annotations such as the different organism dictionaries or the protein family names. In the PC model, chemical entities are also required. In TEES, event extraction is performed using the default system settings and the BioNLP shared task annotations are written into the documents as UIMA annotations.

#### BEL writer

For translation of the annotated BioNLP shared task format to the exact BEL syntax, an appropriate rule set is required. This conversion process is described in detail for GE tasks (38). Table 2 summarizes the main rules for the translation. The standard output for the interaction partners is preferred names in combination with the respective namespace and abundance information [Figure 1, *a(CHEBI:corticosteroid)*]. Abundance functions are mandatory for chemicals and genes within BEL statements. They define the physical forms of the named entities within the relationship. The simple *abundance()* function ‘(in short form: *a()*)’ is used for all chemicals. For the namespaces HGNC, MGI, and RGD, as well as the protein family names (SFAM) and complex names (SCOMP and GOCC), different abundance functions are possible. For entities from these namespaces, the function protein abundance *p()* is chosen by default, but is converted to RNA abundance *r()* for gene expression and transcription events. Furthermore, protein modification events, such as phosphorylation, can be directly converted into BEL terms such as *p(namespace:protein, pmod()).* At least one entity is mandatory for complexes. The ‘+’ indicates that more than one entity can be part of the complex. All events of ‘positive regulation’ in the BioNLP shared task annotations are converted into ‘increase’ statements in BEL. Similarly, all ‘negative regulation’ events are translated to ‘decrease’ relationships. Relations can be expressed recursively; therefore, a BEL term or a full BEL statement can occur as subject or object [referred as B (event) in Table 2**]**.

**Table 2:** Examples of conversion of TEES output (BioNLP Shared Task event annotations) to BEL statements

Example	Sentence snippet	BioNLP		BEL statement
		Event (Name: *trigger word*)	Arguments (Name: *Event or trigger word,…*)	
E1	Earlygrowth response-1 gene expression	Gene_expression: *express*	Theme: *early growth response-1*	p(HGNC:EGR1)
E2	Transfection of Foxp3	Gene_expression: *transfection*	Theme: *Foxp3*	r(HGNC:FOXP3)
		For Gene_expression trigger words ≠ express		
E3	IRF-4 transcription	Transcription: *transcription*	Theme: *IRF-4*	r(HGNC:IRF4)
E4	Phosphorylation of Thr-426 in SREBP1a	Phosphorylation: *Phosphorylation*	Theme: *SREBP1a*, Site: *Thr-426*	p(HGNC:SREBF1, pmod(P,*T,426*))
E5	Degradation of the EGF receptor	ProteinCatabolism: *degradation*	Theme: *EGF receptor*	deg(p(HGNC:EGFR))
E6	Cells treated with LPS secreted similar levels of TNF-alpha	Localization: *secrete*	Theme:*TNF-alpha*	sec(p(HGNC:TNFA))
E7	Surface expression of the LPS receptor complex that comprises TLR4.	Localization: *express*	Theme: *TLR4*, ToLoc: *surface*	surf(p(HGNC:TLR4))
E8	Galangin induced AhR nuclear translocation.	Localization: *translocation*	Theme: *Galangin*, ToLoc: *nuclear*	tloc (p(HGNC:AHR),GOCC:Nucleus)
		For Localization trigger words ≠ secret or express and AtLoc ≠ cell surface or surface		
E9	NF-AT interacts with Foxp3.	Binding: *interacts*	Theme:*NF-AT*, Theme2:*Foxp3*	complex(p(HGNC:NFAT1), p(HGNC:FOXP3))
E10	Galangin induced AhR nuclear translocation.	PositiveRegulation: *induce*	Theme: *E8*, Cause: *galangin*	p(CHEBI:galangin) -> tloc(p(HGNC:AHR), GOCC:Nucleus)
E11	GRK2 decreases early growth response-1 expression.	NegativeRegulation: *decrease*	Theme: *E1*, Cause: *GRK2*	p(HGNC:ADRBP1) -| p(HGNC:EGR1)
E12	STAT4 controls Interleukin 10.	Regulation: *controls*	Theme: *Interleukin 10*, Cause: *STAT4*	p(HGNC:STAT4)−−p(HGNC:IL10)

More details are described in Fluck *et al*. (38).

Currently, the RE tools focus on information given in a single sentence. The flexibility of natural language allows authors to represent information in different ways. Authors must not necessarily describe all information necessary to extract the relationship into one sentence. For these sentences, the integrated RE tools identify incomplete relationships as they are sentence-based. BEL statements without cause or subject are by definition invalid. Therefore, the artificial placeholder entity *a(PH:placeholder)* is introduced when no cause of the relationship is found. This demonstrates the restrictions of the current automated workflow and the need for further manual curation of the TEES output.

For the LibLinear classifier, a positive classified entity pair is encoded in BEL as an “<entity1> association <entity2>” statement. Those relationships enhance the recall of BELIEF but the information is incomplete. There is no subject or object or direction in such a relationship. Unfortunately, in the BioCreative evaluation entity1 is always evaluated as a subject and entity2 as an object. In such a way, some of those relationships are evaluated as false positives. The identification of the predicate and the direction of the relationships as well as the integration of new RE methods and dictionaries are planned for future work to further improve the workflow and to reduce the curation effort accordingly.

### BELIEF Dashboard

The interactive web application BELIEF Dashboard supports the visualization and curation of BEL statements and context annotations automatically extracted by the pipeline. During the implementation of this application, we applied the concepts of user-friendliness and user-interactivity to achieve biocuration efficiency. The BELIEF Dashboard runs in the latest releases of browsers like Firefox, Chrome and Safari (cookies and JavaScript must be enabled). The web application framework Grails^7^ is the basis of the implementation. Grails allows the seamless integration of both Java-based frameworks UIMA and OpenBEL. The UIMA framework is utilized to parse the results of the pipeline. The OpenBEL framework parses and validates the BEL statements. Furthermore, it compares the entered entities and annotations against various OpenBEL namespaces and annotation definitions. An H2^8^ database is used to persist the data.

#### Help pages

The web interface offers help pages to introduce several functionalities of the system. As BEL is not widely used within the biomedical community, a short introduction page is offered to familiarize users with its main concepts. In addition, the process of biocuration with the curation interface is explained in a detailed step-by-step tutorial. Various features of the user interface are described in detail as well.

#### Project and document management system

**Figure 5:**
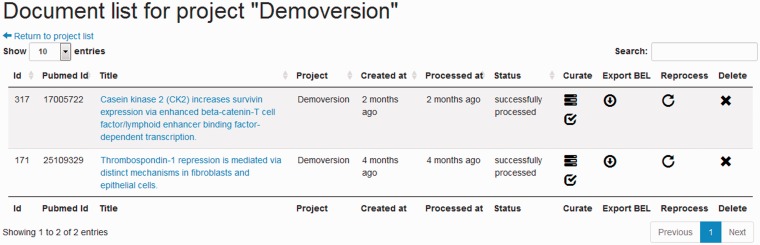
Screenshot of the document management system listing documents for project ‘Demoversion’.

A project in the BELIEF Dashboard represents a curation process project that can contain several documents. A document management function is available, allowing the addition, updating and deletion of any text document (Figure 5). A document can represent an abstract or a full text article. The user can also associate every uploaded document with any of the available projects.

There are three ways to add documents to the BELIEF Dashboard. The first way allows the users to create a single plain-text document and manually fill in citation information such as the PubMed Identifier, title, journal, authors and publication date. The second way is to upload multiple plain-text files to create multiple documents in a single step. The last way is to add abstracts directly from the PubMed database for the provided PubMed article identifiers and automatically fill citation information into the BEL.

An export function enables a BEL document to be downloaded in BELScript format (Figure 5, Column *Export BEL*). BELScript is an internal human readable OpenBEL document format that is used as input for the creation of BEL networks. For further information on how to build networks, we refer the reader to the *Getting Started Guide*^9^ of the OpenBEL framework. The export of the created BEL documents, either for each single text document or for an entire project, is only enabled for syntactically correct BEL statements. The availability of citation information is mandatory. Moreover, only statements and associated context annotations marked as ‘to be exported’ are included into the BEL document. Any syntax error in these BEL statements prevents the entire document from being exported. The errors can be checked and corrected within the interface. For the exported BEL statements, the sentence containing the extracted information is automatically added as ‘summary text’.

#### Document processing queue

Every added text document is introduced into a processing queue. As soon as the BELIEF text mining pipeline becomes idle, it automatically starts retrieving the next unprocessed document from the queue and initiates the processing. The document status changes to ‘processing started.’ When the processing is successfully completed, the results are introduced into the BELIEF Dashboard and the system changes the document status to ‘successfully processed.’ In the case of processing failure, the status changes to ‘processing failed.’

#### BEL curation view

**Figure 6:**
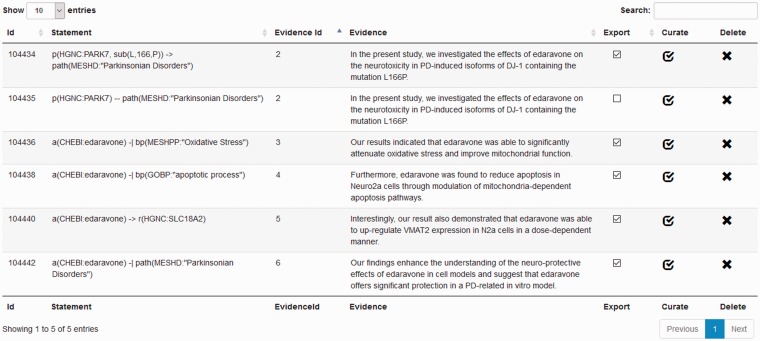
Screenshot of the statement-centric curation view in the BELIEF Dashboard.

Processed documents are available for manual curation. For this purpose, the user can choose between the statement-centric or the evidence-centric curation view to curate a single document. The statement-centric view (Figure 6) lists all detected statements with their evidence in tabular form. In this way, an overview of the results is rapidly provided to the user. The user can delete or mark statements as ‘to be exported’. The link *curate* directs the user to the evidence-centric curation view containing further details and enables manual curation (Figure 4).

The evidence-centric curation view (Figure 4) visualizes extracted BEL statements for each sentence. One sentence in bold is always visualized in the text window in the upper center. To enable a better understanding of the context, sentences surrounding the supporting evidence text are also displayed. Furthermore, in the curation view, all detected concepts found in the inspected sentence are listed in the upper right of the page. Hovering the mouse over the detected concepts highlights the annotated entity in the text window. The concepts consist of BEL namespaces and their normalized names detected by the NER software ProMiner. The identified BEL statements and annotations for the sentence in bold are shown in the lower part of the curation view. This area also allows the modification, deletion or addition of new statements or annotations. The statements and context annotations are automatically validated for correct BEL syntax, valid semantics, available namespaces and reference citation. For invalid objects, a notification box in the lower right hand of the view visualizes the errors and warnings for the user.

Further features are the automatic retrieval of citation information and the concept search, both provided on the right hand side of the curation view. Missing citation information can be added to the document just by defining the PubMed identifier. The information is automatically retrieved by using the web-based PubMed EFetch API. This API allows the querying of data records for specific PubMed identifiers. The concept search in the interface enables the curator to search for missing entities through all namespaces and annotations defined by OpenBEL. Finally, at the lower right of the curation view, all namespaces and annotations are listed and can be browsed by the user.

## Results

In this section, we start with an overview of the performance results for the text mining components within the BELIEF workflow. The BELIEF text mining pipeline as a whole is assessed within the BioCreative V BEL track evaluation environment but outside of the actual challenge. Furthermore, we present the evaluation results for the participation in the BioCreative V Interactive task (IAT). Finally, we discuss the results, outline the lessons learned, and provide some proposals to improve the system.

### BELIEF Pipeline performance

The performances of all single components of the workflow have been reported in several studies. For example, the NER component ProMiner reached an *F*-score value of approximately 80% (recall: 77% and precision: 83%) for human and mouse gene/protein name recognition in previous BioCreative assessments (24, 28). Another assessment of the extraction of BEL relationships revealed rates of correct protein pairs of 60 and 42% for the LibLinear-based classification method and the TEES software, respectively (39). An overall recall rate of 74% was achieved with the combination of both methods. In contrast to the BioCreative assessment, this previous evaluation was performed on sentences with already correctly annotated proteins. Now, with the availability of the BioCreative V BEL track evaluation environment, we can evaluate the BELIEF Pipeline as a whole. In BEL track task 1, based on the provided test set containing 100 sentences, the systems were asked to extract the corresponding BEL statements automatically. These sentences were sent in command line mode to the BELIEF system. No prior training of the text-mining pipeline was performed. The only evaluation-related changes we made were as follows:

– inclusion of the selected GO biological processes in the RE process;
– removal of all relationships with non-BEL-track namespaces in a subsequent process.

Otherwise, the pipeline was used without any further adaptations.

Table 3 shows the detailed evaluation results, covering precision, recall and F-score for the different levels. The scores provided by the BEL track evaluation are for the correctness of full BEL statements. Since the extraction of completely correct BEL statements is difficult to achieve, not only the full statement (S) but also the correct recognition of the normalized entities (T), the functions (FS and F) and the relationships (RS and R) was measured. A function (F) is defined by a regulatory function name and one argument. In the case of relation (R) a predicate and two arguments are needed. The secondary classes (FS and RS) mean that a maximum of one argument is missing. For a detailed description of these classes used in this task, we refer to Rinaldi *et al*. (7). For full statement recognition, our system achieved an F-score of 30.8% with precision of 59.1% and recall of 20.8%. Considering the complexity of the task, these are consistent with state of the art for extraction of complex interactions. Correct relationships, ignoring functions, could be extracted with an *F*-score of 43.1% (Relationship). At least partially correct relationships could be extracted with an *F*-score of 64.9% (Relationship-Secondary). At this level, only the subject or the object together with the correct predicate or alternatively, both subject and object have to be correct.

**Table 3:** Test set prediction results for the several classes of BioCreative V BEL track task 1

Class	Precision	Recall	*F*-score
Term (T)	**81.34**	72.67	76.76
Function-Secondary (FS)	**66.67**	39.29	49.44
Function (F)	**51.16**	33.33	40.37
Relationship-Secondary (RS)	56.65	**73.76**	64.09
Relationship (R)	**67.37**	31.68	43.10
Statement (S)	**59.15**	20.79	30.77

The classes represent the different structural levels of a BEL statement. The description of the classes and several examples can be found in Section 3.1 and in Rinaldi et al. (7). (Note. The secondary classes stand for partially correct information.)

At the function extraction level, the functions in general were found with an F-score of almost 49.4% (Function-Secondary). In this case, only the correct function term such as *act()* or *pmod()* has to be found in a BEL statement. The correct entity association is not taken into account. When correct association of the function with the valid entity (Function) was considered, the F-score dropped to 40.4%. Most recall errors at the function level involved missing activity function assignments. The activity function is assigned when a protein is in an active state (e.g. an activated kinase). Currently, the BELIEF system does not introduce any activity functions into BEL statements. When measuring NER performance within the BELIEF extraction, F-score values of 76.7% were reached. Compared with the previous gene and protein NER evaluation alone, that constitutes a drop of around 4%. This drop in performance is not surprising since for the BioCreative V BEL track two conditions have to be met: correct recognition of the entity and the participation in an extracted relationship. Overall, the system achieved higher precision and lower recall for all levels except for the secondary relationship level.

Some evaluation examples based on the BioCreative sample set are shown in Table 4. These examples give an impression of the success and failure of the automatic extraction and the curation effort. In the first example, two relationships are predicted by the BELIEF system. The first one is correct, the second one completely wrong. Nevertheless, all entities were normalized correctly and are already in correct BEL expressions. For correction, the curator has to perform two copy and paste steps: (1) copy ‘*a(CHEBI:"chondroitin sulfate")*’ and paste it as a subject in a new statement field and (2) copy ‘*-| p(HGNC:FUS)*’ next to the new subject. Furthermore, the curator exports only correct statements. All other statements that are not marked as exported are omitted later by the BELIEF Dashboard.

**Table 4:** Some evaluation examples based on the sentences derived from BioCreative V BEL track sample set

**Example 1**		
The nuclear protein pigpen has an affinity for carbohydrate structures a carbohydrate-binding domain resides in the C terminus of the molecule and can be preferentially inhibited by saccharides, most notably N-acetyl-d-galactosamine and chondroitin sulphate.		
Type	Result	BEL statement

Gold	–	a(CHEBI:"N-acetyl-D-galactosamine") -| p(HGNC:FUS)
	–	a(CHEBI:"chondroitin sulfate") -| p(HGNC:FUS)
BELIEF	True positive	a(CHEBI:"N-acetyl-D-galactosamine") -| p(HGNC:FUS)
	False positive	a(CHEBI:"N-acetyl-D-galactosamine") -| a(CHEBI:"chondroitin sulfate")

**Example 2**		
60 or 90 μM galangin induced AhR nuclear translocation in both cell type (Figure 11A, lanes 7, 8; Figure 11B, lane 6).		

Type	Result	BEL statement

Gold	–	a(CHEBI:galangin) -> act(p(HGNC:AHR))
BELIEF	True positive	a(CHEBI:galangin) -> tloc(p(HGNC:AHR))

**Example 3**		
In the absence of CdCl2 pre-treatment, ionizing radiation increased both expression and phosphorylation of c-Jun in MRC5CV1 cells but not in AT5BIVA cells.		

Type	Result	BEL statement

Gold	–	bp(GOBP:"response to ionizing radiation") -> p(HGNC:JUN,pmod(P))
	–	bp(GOBP:"response to ionizing radiation") -> p(HGNC:JUN)
BELIEF	False positive	p(PH:placeholder) -| (a(CHEBI:"cadmium dichloride") -> p(HGNC:JUN,pmod(P)))
	False positive	p(PH:placeholder) -| (a(CHEBI:"cadmium dichloride") -> p(HGNC:JUN))
	False positive	p(HGNC:JUN) −− a(CHEBI:"cadmium dichloride")

**Example 4**		
The sensitivity to Fas-induced cell death was reduced in HGF transfectants, which was reversed by the presence of anti-HGF antibody		

Type	Result	BEL statement

Gold	–	p(HGNC:HGF) -| (act(p(HGNC:FAS)) -> bp(GOBP:"cell death"))
BELIEF	Partly true positive	p(PH:placeholder) -| (p(HGNC:FAS) -> bp(GOBP:"cell death"))
	Partly true positive	p(HGNC:FAS) −− bp(GOBP:"cell death")
	False positive	p(HGNC:HGF) −− bp(GOBP:"cell death")

Every example contains a sentence with the gold standard and predicted BEL statements. In addition, the results of the predicted BEL statements are provided.

In the second example, the BEL statement is the correct extraction of the relation expressed in the sentence. In contrast, the gold standard contains the activation function of *HGNC:AHR* (expressed as *act()*) instead of the translocation function *tloc()*. For a biologist, it is well known that the translocation of AHR to the nucleus activates AHR. Especially in the training data, a large number of statements containing biological interpretation are included. In the test set, most of those interpretations are removed.

Missing interpretation of negations or recursive relationships in the BELIEF extraction is another error source. Examples 3 and 4 show an example of such cases. In Example 3, from the text: ‘In the absence of CdCl2 pre-treatment’ a negative relation ‘*a(PH:placeholder) -| a(CHEBI:"cadmium dichloride")*’ is extracted. BELIEF supplements missing subjects by the artificial entity ‘a(PH:placeholder)’ (“BEL writer” section). The BioCreative evaluation framework accepts those placeholder entities without causing errors. In such a way, partial relations can be proposed to the curator. For missing predicates the relationship type association (‘−−’) is used as a placeholder. Association predictions are generated by the LibLinear classifier. Overall, this classifier provides more recall for the relations but offers no specific relationship type. In the case of Example 3, it provides a false prediction. However, despite all these placeholders, the system failed for Example 3 because the correct subject could not be identified. BELIEF missed the GO biological process *response to ionizing radiation*. Missing entities, or in some cases, additional entities are the main reason for system failure. In these cases, only parts of the detected relations can be used and missing entities must to be looked for and added manually by the curator. The curator can use the namespace search within the curation interface to search for the correct entity.

We note that the predictions in Example 3 do not support a curator substantially. In contrast, Example 4 shows that these partial relationships are of great value for curation. The placeholder in the first extraction could easily be replaced by ‘*p(HGNC:HGF)*’provided in the last predicted statement. Only the second predicted statement does not give any additional relationship information.

Overall, the evaluation results are promising but clearly show room for improvement such as removing redundant information. The interpretation of the relations and merging of extracted statements is a step that will be tackled next to further improve the system performance.

### Curation interface testing in BioCreative V interactive task (IAT) track

The IAT involves the performance of a formal evaluation of the text mining systems for a specific biocuration task. The systems are evaluated for their performance (time-on-task and accuracy of text mining-assisted curation compared with manual curation) and a subjective system usability measure via a user survey (19).

Seven curators were invited by the task organizers and provided access to the BELIEF Dashboard, among whom five fully participated in this task. Among these participants, one was an experienced BEL curator and two had some basic knowledge of BEL and BEL coding. The other two individuals who fully participated were database curators with no prior knowledge of BEL. None of the participants knew about the functionality of the BELIEF Dashboard. Before the assignment of the tasks, the participants were asked to work through the provided tutorials about BEL and the functionality of the dashboard. In the next step, they were subjected to a three-document-based training session to learn the curation process and important characteristics of the system. After this initial session, the organizers conducted a first survey with the participants to test their understanding of the system.

In the main test phase, the curators were randomly divided into two groups. A total of 20 PubMed abstracts were provided to the curators. These documents were selected because they contained a large number of relationships that could potentially be extracted. We divided the corpus in two sets (Set 1 and Set 2) each containing 10 documents. We assigned Set 1 to Group 1 for the text mining-assisted curation and to Group 2 for manual curation, and vice versa for Set 2 (Table 5). Although, all subjects were asked to invest one hour each in the text mining-assisted and unassisted curation tasks, some curators invested more time on their own initiative.

**Table 5:** Usage of the document sets by the two groups

	Assisted curation	Manual curation
Group 1 (*n* = 2)	Set 1	Set 2
Group 2 (*n* = 3)	Set 2	Set 1

#### Annotation guidelines

To guide biocuration and create consistent high-quality annotations, well-defined annotation guidelines play a crucial role. However, for such a task, the guidelines should be short and easy to follow. The following describes the annotation guidelines for curation with the BELIEF Dashboard.

- Obtain an initial overview of the automatically generated BEL statements in the statement-centric curation view.
- Curate and add missing statements in the evidence-centric curation view. Activate the export flag only for these statements. It is not necessary to delete statements: use only the most appropriate statements to curate and export.
- If possible, replace the association relationship (–) with a more precise relationship type. The association relationship is detected by the simple LibLinear-based classification method.
- Add and curate the corresponding context annotations (e.g. experimental parameters and context) to the statements.
- Correct the syntax and semantic errors and provide only valid statements and context annotations.
- After finishing, export the BEL document.

The annotation guidelines for manual curation are as follows:

1. Download the pre-defined BEL document templates from the training area under the BELIEF Dashboard. These documents already contain definitions for OpenBEL namespaces and citation information.
2. Add the relevant BEL statements together with the evidence information to the BEL document.
3. Add the relevant context annotations to the BEL statements.
4. If possible, compile the document with the OpenBEL framework to identify and correct syntax and semantic errors.

#### Biocuration results

**Figure 7:**
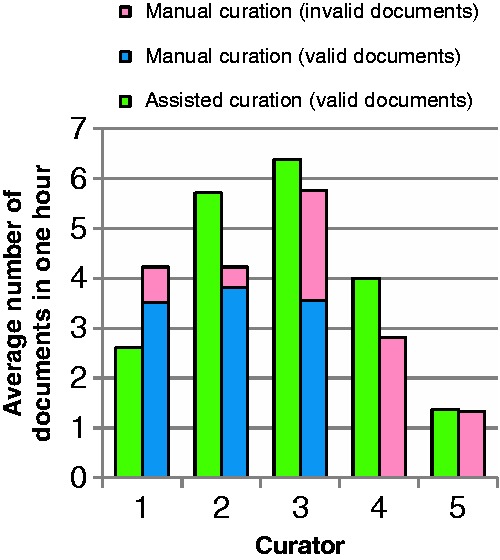
Average number of documents curated by curators in 1 h through assited curation using BELIEF Dashboard and through manual curation. The documents with BEL syntax errors are shown here as invalid. The curators are ordered according to their experience of BEL curation.

Most of the participants did not annotate all of the provided documents. An overview of average number of curated documents for each curator for both curation types is shown in Figure 7. The participants are ordered according to their experience of BEL curation. Curator 1 had the most BEL curation experience, curators 2 and 3 had some experience and curators 4 and 5 had no prior knowledge of BEL. Out of all of the participants, only curator 2 was able to finish the task of curating 10 documents. On average, 5.0 documents were annotated with text mining support (Figure 7, green bars and Table 6) and 4.8 documents by unassisted curation (Figure 7, blue bars and Table 6). Only curator 1, who had the most experience of unassisted BEL coding, curated more documents in the unassisted mode. The testers with no prior BEL knowledge curated the fewest documents in both settings.

**Table 6:** Resulted documents and BEL statements through assisted curation using BELIEF Dashboard and through manual curation

	Documents		BEL Statements	
	Assisted curation	Manual curation	Assisted curation	Manual curation
Syntactically valid	25	16	243	159
Syntactically invalid	0	8	0	27
	25	24	243	186

The invalid category represents the documents and BEL statements with syntax errors.

Table 6 describes the curation results for assisted and manual curation. All BEL documents curated within the BELIEF Dashboard were syntactically correct and contained altogether 243 BEL statements. Among the documents curated without text mining assistance, eight documents (33.3%) were syntactically invalid. In summary, altogether 186 BEL statements were created in this set, 27 of which were invalid, resulting in a rate of invalid statements of 14.52% overall.

**Figure 8:**
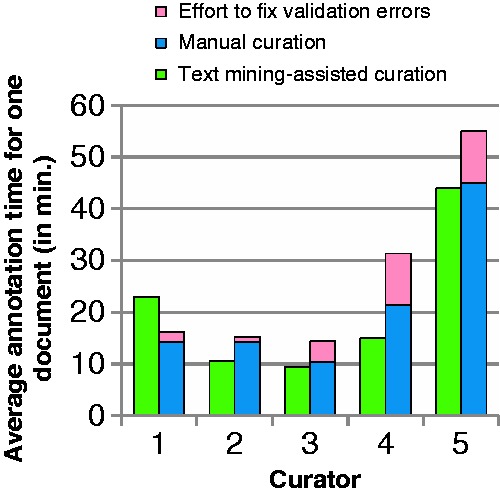
Time usage by curators and curation type.

The times required by the testers for curation with the BELIEF Dashboard and for manual curation are shown in Figure 8. Only the most experienced tester, who was used to unassisted BEL coding in particular, needed more curation time with the BELIEF Dashboard than without text mining assistance. For all of the other curators, the curation time was less than for unassisted curation, even though more BEL statements (23% more statements) were curated. It is also necessary to take into account the additional effort that has to be expended on the manual curation of several invalid documents. The following tasks are necessary to clean up these invalid documents: (1) compile the document with the OpenBEL framework, (2) localize errors in the BEL document, (3) fix these errors and (4) repeat until all errors are fixed. This time was added to the average curation time for the participants (Figure 8, pink bars). Curator four with no prior knowledge of BEL gained the most from the BELIEF support and needed 12 min on average, compared with over 20 min for unassisted coding. Accounting for the additional syntax correction time, the time spent was almost halved. The last participant struggled with BEL coding in general and needed the most time for the curation in both settings.

#### Curator survey results

After finishing the curation task, a survey consisted of a 10-item questionnaire was conducted with the participants. These questions are derived from the *System Usability Scale*^10^ (SUS) method, which measures the perception of usability and learnability of a software product. From the 10 questions, eight measure the usability. The remaining two questions quantify the learnability. The score ranges from 0 to 100 and a score of 68 (not a percentage) is considered to be the average. The BELIEF Dashboard achieved a calculated average SUS score of 67. The system reached average SUS scores of 67 and 65 for usability and learnability, respectively.

Within the survey, the participants were also asked to comment on the questions, if they wished. Some of these comments are listed below:

- *Complexity of BEL:* ‘The complexity was in the BEL language itself; the BELIEF system actually made it easier to start understanding how interactions were encoded’ and ‘The system is very easy to learn for a user who is already familiar with BEL’.
- *Concept search:* ‘More tools could be added to help the curator, such as a search by term, not only by namespace + term. It would also be useful to know when the available namespaces that are available were updated in the case that there is a missing term; one can know if it is a bug or an update issue’.
- *Named entity detection:* ‘In particular, the pre-selected protein identifiers were immensely useful (which I only found out when I tried to find them by hand)’.
- *Relationship extraction:* ‘It was cumbersome to sort through the less relevant results, but overall the system was easy to use and the disambiguation was helpful’.
- *BEL validator:* ‘No way of suggesting how to correct mistakes or any correct examples’.
- *Team support:* ‘I found it really interesting to participate in this task; people were extremely helpful and fast in answering the questions I had’.

## Discussion

In this article, we have presented and evaluated the BELIEF workflow that was developed to accelerate information extraction from the biomedical literature. It consists of a text mining pipeline and a web-based curation tool. The BELIEF curation interface uses the text mining pipeline to categorize biological knowledge in the form of causal and correlative relationships. These relationships are encoded in BEL statements and their context annotations that can be reviewed and curated by expert biocurators directly in the user interface.

The BELIEF system was evaluated on two different levels with the support of the BioCreative V assessments. The quality of the BELIEF information extraction was tested using the evaluation environment provided by the BEL track. Furthermore, usability aspects were evaluated in the BioCreative V IAT.

The performance measurement for the BELIEF Pipeline showed that the integrated text mining workflow generates competitive results (Table 3). Compared with the outcome of the BEL track task 1, BELIEF generated the highest F-score for fully correct BEL statements (30.8 versus 20.2% for the best BioCreative evaluated system). In addition, for the recognition of functions, results equivalent to those of the best BioCreative participant were achieved. For the assignment of correct protein modification information and the recognition of complexes, we achieved an F-score of 40.4%, 7.8% higher than that of the best BioCreative participant. In contrast, at the Function-Secondary level, the best BioCreative F-score was 54.6% and BELIEF reached a score of 49.4%. In both cases, BELIEF has a higher precision (51.2 and 66.7%, respectively). At the RE level, the BELIEF system performed second best. BELIEF recognized relationships with an F-score of 43.1%, whereas the best BEL track participant achieved a score of 49.2%.

Table 4 provides some typical automatic extractions and the gold statements for comparison. Missing entity recognition, especially for biological processes but also for the other classes are a main source for error. In addition, the current BELIEF system lacks a filter for the removal of redundant statements or any post-processing for the interpretation or merging of statements. Furthermore, the LibLinerar classifier generates only undirected relations. In these cases, the subject and object are assigned by chance. This is an area where an additional post-processing step could help in assigning subjects/objects that are more correct and also predict a relationship type. Furthermore, prior knowledge could be applied to resolve ambiguities (i.e. kinases perform phosphorylations). The inclusion of inference methods is necessary to permit a conversion of recursive relationships such as decrease of decrease leads to an increase. It remains to be evaluated where the limitations of such automatic inference methods are. Similarly, the differentiation of direct versus indirect relationships and causality versus correlation are open questions. In the future, we plan to extend the text mining workflow by adding rule-based tools and/or improved machine learning models for RE in order to improve these results towards the above mentioned open issues. An important pre-condition for method improvements is the availability of proper training data and evaluation environments. The BioCreative V task 4 provides both in a first version. We hope to generate more training data by providing BELIEF to curators and storing automatically generated and curated statements.

The second evaluation was carried out through participation in the IAT. It concerns the usability of the developed BELIEF Pipeline and the curation interface within the BELIEF Dashboard. A pre-condition of any assisted or manual BEL curation was knowledge about the syntax and concepts of BEL. For two curators who had no previous experience of BEL, learning a new and complex language was challenging given the limited time that they had to perform the task. Although the surveys showed that the provided documentation (help pages) was helpful to explain BEL, further sophisticated e-learning techniques such as screencasts and webinars could prove even more useful to introduce the structure of BEL and the main functionalities of the system in a simple and effective manner.

The statement-centric view in the BELIEF curation interface provides a fast and comprehensive overview of automatically extracted statements. The evidence-centric view shows the evidence sentence with the associated named entities, allowing rapid review and modification of BEL statements and their context annotations. The accuracy of named entity detection was considered to be very helpful by all participants in the curation task. Overall, curation speed could be increased with the support of BELIEF and 23% more statements were generated by text-mining-assisted curation. Nevertheless, we had expected more time saving on the curation task when compared to a previous evaluation where the curation exercise was executed with experienced BELIEF users who also had prior knowledge of BEL (26). One reason for this is the lack of an e-learning environment to introduce BEL and BELIEF properly to the users, as mentioned previously. Another problem with the current system is to some extent the large number of BEL statements that the system proposes. The reasons for this include that, in some cases, the current RE workflow outputs a number of incomplete statements leading to a larger number of extracted statements. Trained BELIEF curators use those incomplete statements to assemble correct statements and mark them for export. Unmarked statements are ignored by the system in the later export phase. In the BioCreative V IAT, some participants did not follow the guidelines correctly and did not only select statements for export, but removed all false BEL statements from the system. This increased the curation time and reduced the time-on-task performance for assisted curation. As mentioned above, we plan to improve the RE in order to reduce the overall number of statements. The evaluation environment developed in the course of the BEL track will help us in further optimizing the text mining output.

The integrated BEL validator component was used to validate the added or modified BEL statements with the context annotations in the curation interface. This component generates syntax and semantic error messages for invalid BEL statements. As a result, BEL documents exported by the system are always syntactically correct. In the feedback from the survey, the biocurators criticized the complexity of these messages. It is most likely that the performance of curation could improve if these messages were more instructive. In future versions, an additional help page introducing various categories of error and instructions on fixing the invalid statements will hopefully lead to a better understanding of the messages. Nevertheless, to correct invalid BEL statements, use of the BEL framework is even more time-consuming than using BELIEF. In the presented evaluation, we only measured the time OpenBEL experts needed to correct the syntactically invalid statements. Without experience, the correction time would be much longer. Therefore, from the perspective of usability, it makes perfect sense to include the validation process directly in the interface.

Overall, the users rated their experience with BELIEF as positive. All users shared the opinion that BELIEF speeds up the curation of BEL statements. The SUS method, which measures the usability and learnability, revealed that the interface is fairly usable, but still has room for improvement. It has been stated explicitly that the SUS method lacks diagnostic information (40). It does not provide any details about which system components can be improved and how. Therefore, it is necessary to consider further usability analysis questionnaires and tools for a detailed examination of the system.

## Conclusion

We presented a user-friendly and web-based curation interface that incorporates a semiautomatic knowledge extraction workflow to support network building for systems biology. The text mining pipeline performs well when compared with the BioCreative V BEL track evaluation. It allows biocurators to extract knowledge from the biomedical literature and curate causal and correlative relationships encoded into BEL. During the BioCreative V IAT track, the curation interface was evaluated based on its performance and a user survey. We showed that the BELIEF Dashboard increased the curation efficiency when compared with manual curation. Furthermore, the task helped to identify various aspects of the interface that were useful for the curation and revealed important issues for future improvement.

## Footnotes

1 http://wiki.openbel.org/.

2 http://www.biocreative.org/.

3 http://uima.apache.org.

4 http://commons.apache.org/proper/commons-daemon/jsvc.html.

5 Representational State Transfer, JAX-RS.

6 http://2013.bionlp-st.org/.

7 https://www.grails.org/.

8 http://www.h2database.com/.

9 http://wiki.openbel.org/display/BFD/Getting+Started+-+Getting+Started+Guide.

10 http://www.measuringu.com/sus.php.
